# *Genista cephalantha* Spach. protects against acetaminophen-induced liver failure via preserving the glutathione redox system, reducing inﬂammatory response, and inhibiting hepatocyte death in rats

**DOI:** 10.22038/IJBMS.2024.73804.16040

**Published:** 2024

**Authors:** Boulkandoul Ramzi, Ameddah Souad, Chebbah Kawthar, Erenler Ramazan, Mekkiou Ratiba, Benayache Samir, Benayache Fadila, Menad Ahmed

**Affiliations:** 1 Laboratoire de Biologie et Environnement. Université Frères Mentouri Constantine 1, Algérie; 2 Unité de Recherche, Valorisation des Ressources Naturelles, Molécules Bioactives et Analyses Physicochimiques et Biologiques. Frères Mentouri Constantine 1, Algérie; 3 Research Laboratory Practice and Research Center, Igdir, University Igdir, Turkiye

**Keywords:** Acetaminophen, Cell death, Hepatoprotection, Inflammation, LC-MS/MS, Necroptosis, Necrostatin-1, Oxidative stress

## Abstract

**Objective(s):**

The current study was conducted to assess the protective mechanisms of *n-*BuOH fraction from the aerial part of *Genista cephontala* (BEGC) on APAP-induced liver injury compared to necrostatine-1 (Nec-1).

**Materials and Methods:**

A model of APAP-induced hepatotoxicity was created in male rats by injecting a single dose; 1000 mg/kg APAP, the protective effect was performed with (200 mg/kg; 10 days) BEGC compared to Nec-1, (1.8 mg/kg).

**Results:**

BEGC or NeC-1 pretreatment significantly abolished impaired effects in APAP-rats, by decreasing the generation of TBARS and ROS in mitochondrial and cytosolic fractions and maintaining liver function activities. A marked response was observed in the levels of both GSH and GSH-system enzymes in liver homogenates and mitochondrial fractions to BEGC. BEGC/ Nec-1 successfully regulated the inflammatory mediators (IL-β, TNF-α, HMGB1, and acHMGB1) and MPO levels. During APAP treatment, no caspase-3 or -8 activity was detected, and the level of fk18; M30 was higher than the levels of cck18; M65. Moreover, RIPK3 and MLKL levels were increased in the APAP group. These results suggested that necroptosis predominates during the APAP liver injury model. Interestingly, these necroptotic factors were significantly down-regulated by BEGC treatment. Both biochemical and histopathological findings were consistent with each other.

**Conclusion:**

From all these findings, the hepatoprotective effect of BEGC could be due to the abundance of polyphenols identified by LC-MS/MS analysis, as well as the synergistic interactions of all contents.

## Introduction

Acetaminophen (APAP) toxicity is known to include multiple steps and signaling pathways (1). Mechanically, APAP-induced hepatotoxicity initiates with an excessive generation of the reactive metabolite, NAPQI, that decreases GSH level and impairs mitochondrial functions, resulting in overproduction of mitochondria reactive oxygen species (ROS) (2). Liver tissue homeostasis is ensured by an equilibrium between cell growth and cell death (3). The type and mechanism of APAP-triggered hepatocyte death are still matters of debate (4). Programmed cell death necrosis and/or apoptosis have been demonstrated to participate in APAP-induced liver damage; necroptosis which represents one of the programmed cell death involved in APAP-hepatotoxicity, has not yet been studied in detail (5). A good understanding of the mechanisms of APAP-hepatotoxicity allows the identification of therapeutic pathways and the development of a more reactive antidote than NAC, previously considered one of the most important antidotes to APAP (4). Therapeutic options for treating APAP hepatotoxicity remain limited (6). Recently, necrostatin-1 (Nec-1) has been considered a promising therapeutic approach in a number of deadly situations (7). Currently, research focuses on finding new drugs by investigating the active effects of medicinal plants and natural compounds (8).

In Algeria, the genus *Genista* (Fabaceae) is represented by 25 species of which 11 species are endemic (9). The *Genista* genus is characterized by the presence of various bioactive compounds, including the predominant flavonoids (10). The genus is characterized by the presence of various bioactive compounds, including the predominant flavonoids (10). Traditionally, *G. cephalanta* has a wide application, however, the scientific evidence is lacking. Taking into account that leaves and flowers of *n*-BuOH fraction from *G. cephalanta *(BEGC) grown in eastern Algeria have never been screened for hepatoprotective effect, the current study aimed to investigate the potential hepatoprotective effect of BEGC anti-APAP-induced liver damage compared to the Nec-1 agent and explored its possible underlying mechanisms; we are also aiming to screen the different chemical compositions to widen knowledge on secondary metabolites from *G. cephalanta* (M’sila region; Algeria) using LC-MS/MS analyses.

## Materials and Methods

***Chemical reagents***

Acetaminophen (APAP; 98.0% HPLC); Necrostatin-1 (Nec-1); reduced glutathione (GSH); glutathione reductase (GR); 5,5-dithiobis-2-nitrobenzoic (DTNB); 1-chloro-2,4-dinitrobenzene (CDNB); thiobarbituric acid (TBA); ethylene glycol bis(2-aminoethylether)-N,N,N′,N′-tetraacetic acid (EGTA); hexadecyltrimethyl ammonium bromide (HTAB); *O*-dianisidine; dichlorofluorescin diacetate (DCFDA); bovine serum albumin (BSA); dimethyl sulfoxide (DMSO); phenylmethylsulfonyl fluoride; pepstatin; and leupeptin were purchased from Sigma-Aldrich Chemical Co (StLouis, MO, USA).

Rat tumor necrosis factor-𝛼 (TNF-𝛼); rat interleukin- β (IL-β); nuclear high mobility group box1(HMGB1); hyperacetylated (acHMGB1); caspase-3 and caspase-8 (caspase specific fluorometric assay kits purchased from Calbiochem Inc. (Darmstadt, Germany). Receptor-interacting protein kinase (RIPK3); mixed lineage kinase domain-like protein (MLKL); cytokeratin 18 full-length protein (fk18; M30); caspase-cleaved keratin 18 (cck18, M65); (CK 18-M30, CK 18-M65) were purchased from Protein Tech Group, Inc (Rosemont, IL, United States). Standards for LC-MS/MS analysis (chlorogenic acid, naringenin, *p*-hydroxybenzoic acid, 4OH-benzaldehyde, gentisic acid, 

*p*-coumaric acid, quercetin, gallic acid, rosmarinic acid, rutin, oleuropein, and caffeic acid) were purchased from Extra synthese (Genay, France). The solvents and/or reagents were purchased from Sigma Chemical Co. (St. Louis, MO, USA).

***Plant extracts preparation***

Aerial parts of *G. cephalantha* Spach. were harvested during flowering in the Mesila region in eastern Algeria and authenticated by Dr. D. Sarri (Department of Biology, University of Mesila, Algeria) based on Quezel and Santa (9). A voucher specimen (FGC15/06/19 has been filed in the Herbarium of the VARENBIOMOL unit research, University of Constantine1. Air-dried aerial parts (leaves and flowers, 2000 g) of *G. cephalantha* were powdered and macerated at room temperature with MeOH-H_2_O (70:30, v/v) three times (24 hr for each time). After filtration, the solution obtained was successively extracted with solvents of increasing polarities: chloroform (CHCl3), ethyl acetate (EtOAc), and n-butanol (*n*-BuOH)). The organic layers were dried with anhydrous Na_2_SO_4_, filtered, and concentrated under vacuum at room temperature to obtain CHCl_3_ (2. 20 g, EtOAc (4.74 g,) and *n*-BuOH (25.55 g) extracts. A part of *n*-BuOH extract of *G. cephalanta* (BEGC) was used for the hepatoprotective study.

***Liquid-chromatography tandem mass spectrometry (LC-MS/MS)***

LC-MS/MS analysis of *n*-BuOH of *G.* *cephalantha* was performed using a Thermo Scientific Dionex Ultimate 3000 -TSQ Quantum with a Thermo ODS Hypersil column (250×4.6 mm, particle size of 5 µm). The electrospray ionization (ESI) source of the mass spectrometer was operated in both positive and negative modes. The spray voltages for positive and negative polarities were 4000 and 2500 V, respectively.

***Animals***

Swiss albino rats (230±2 g) were used in the experiment. Standard laboratory conditions were a temperature of 25±2 ^°^C and a 12 hr light/12 hr dark cycle. The animals were fed standard rodent pellet diet and water *ad libitum*. The *in vivo* experimental protocol was agreed upon by the Institutional Project Committee (PRFU, D01N01UN250120190002). The experimental procedures adopted in this study required adherence to the Guidelines for Reporting Animal Research (11).

***Acute toxicity evaluation of BEGC***

Acute toxicity study was achieved as reported by Organisation for Economic Co-operation and Development guidelines (OCDE) No.425. Male Swiss albino rats were divided into 4 groups, each having 3 rats. BEGC was administered orally as a single dose to rats at different doses of 250, 500, 1000, 1500, and 2000 mg/kg b.w. The animals were monitored periodically for signs of toxicity, particularly body weight variation, diarrhea, changes in general behavior, physical appearance, and mortalities within 24 hr and then daily for 14 days.

***Hepatoprotective effect of BEGC***

*Experimental design*

To evaluate the hepatoprotective effects of BEGC against the hepatotoxicity induced with APAP rats were grouped into four groups (12 rats per group): 

Group 1 (control-group): served as a control, was orally treated with the vehicle (DMSO/H_2_O) for ten days. 

Group 2 (APAP-group): administered intraperitoneal (IP) a single dose of APAP (1000 mg/kg) dissolved in DMSO/H_2_O after ten days’ administration of vehicle (12).

Group 3 (BEGC-group): received orally BEGC (200 mg/kg) for ten days. Group 4 (Nec-1–group+APAP): received IP Nec-1 (1.8mg/kg)(13) dissolved in DMSO/H2O for ten days before APAP administration. 

Group 5 (BEGC-group+APAP): received BEGC orally (200 mg/kg) for ten days, before APAP administration.

At the end of treatment on day 11, all animals were killed by decapitation. A fragment from the removed liver was handled for the preparation of cytosolic and mitochondrial fractions, which were conserved at -80 ^°^C until used for various biochemical assays, the rest for cell death and proinflammatory parameters.

***Measurement of the hepatic function biomarkers***

The serum analysis of various liver markers (AST, ALT, LDH, and γ-GT activities) was measured using assay kits. The rest of the serum was frozen at -80 ^°^C until measurement of biochemical parameters.

***Preparation of cytosolic and mitochondrial fractions***

The preparation of the cytosolic fraction and the hepatic mitochondrial fraction was based on differential centrifugation (14). Part of the liver was quickly immersed in ice-cold isolation buffer (mannitol 220 mmol/l, sucrose 70 mmol/l, morpholinopropane sulfonic acid 5 mmol/l, pH 7.4). The minced tissues were homogenized with additional homogenization media (isolation buffer plus EGTA 2 mmol/l) 10 volumes (wt/vol) in a Potter Elvehjem homogenizer with a loose-fitting Teflon pestle (four strokes). The homogenates were then centrifuged for 10 min at 700 x g. The supernatants were collected and centrifuged again for 10 min at 10,000 × g, 4 ^°^C. The supernatant was used for the assessment of the cytosolic anti-oxidant status, the pellet was resuspended in isolation buffer and centrifuged twice for 10 min at 7000 × g (cycle washing was repeated three times for further purification of mitochondria, then suspended in buffer). Liver mitochondrial fraction was used to estimate oxidative status.

***ROS quantification***

Autofluorescence 2’,7’-dichlorofluorescin diacetate was utilized for ROS estimation, and 100 µl of tissue homogenate/ mitochondrial fraction, were incubated with DCFDA (final concentration: 10 µM) diluted 1:200 in Tris-HCl buffer. After 15 min of incubation at 37 ^°^C, fluorescence was determined with excitation of 488 nm and emission of 525 nm using a fluorescence plate reader (15).

***Analysis of liver oxidative stress status parameters***

*Malondialdehyde (MDA) assay*

MDA was estimated in the cytosol and the mitochondrial fraction, by measuring TBARS according to Ohkawa *et al.* (16). ROS was evaluated spectrophotometrically at 535 nm and the amount of TBARS was calculated using a molar extinction coefficient of 1.56 x 10^5^ M^-1^cm^-1^; the results were expressed in µmol/mg protein.

***Measurement of glutathione and glutathione-metabolizing enzymes***

Estimation of reduced glutathione (GSH) levels was based on the reaction of GSH with DTNB producing a yellow-coloured complex estimated at 412 nm according to the method of Sedlak and Hanus (17). GSH levels were calculated using an extinction coefficient of 13600 moL/cm, values were expressed in nmol/mg protein.

Glutathione-S-transferase (GST) activity was assayed by quantifying the conjugate glutathione resulting from the conjugation of CDNB with GSH following the method of Habig *et al.* (18). GST activity was monitored at 340 nm for 3 min. Enzyme activity was expressed in U/mg protein; one unit of GST activity was defined as µmoles CDNB conjugate formed/min/mg protein using a molar extinction coefficient of 9.6×103 M^-1^cm^-1^. Glutathione peroxidase (GPx) activity was determined by the method of Rotruck *et al*. based on the degradation of H_2_O_2_ in the presence of GSH (19). Enzyme activity was expressed as U/mg protein; one unit of GPx activity was defined as nmoles GSH/mg protein. Glutathione reductase (GR) activity was assayed by measuring NADPH oxidation at 340 nm using oxidized glutathione as a substrate (20). The results were expressed as U/mg protein (U: µmol NADPH oxidized/min). Protein estimation in liver tissue was assayed by Lowry’s method (21) using BSA as standard.

***Measurement of cell death markers***

*Measurement of caspase-3 and caspase-8 activities*

For the caspase assays, 50 µl of the diluted plasma for each sample and 50 μl caspase-3 and caspase-8 activities were determined using various caspase-specific fluorometric assay kits. Specific substrate conjugate was added to the reaction mixture to start the reaction, which was recorded immediately using a multi-well fluorescence plate reader at excitation/emission wavelengths of 400/505 nm at 28 °C (22). The increase in the relative fluorescence units (RFU) was recorded as a kinetic plot over time. The caspase activity was expressed as RFU/hr/µl.

***Plasma CK18–measurement (M65 or M30)***

The degree and form of hepatocyte death were analyzed by measuring the CK18 concentration of plasma.CK18-Asp396 levels were assessed using the M30 Apoptosense ELISA (ccCK18) and for total soluble CK18 using the M65-ELISA, according to the manufacturer’s instructions, the absorbance was measured in a microplate reader at 450 nm. The concentration of ccCK18 and total CK18 in serum is presented as U/l.

***Measurement*** *of* ***necroptosis markers***

RIPK3 and MIKL levels were measured in duplicate in 1:40 diluted cytosol samples using a commercially available assay ELISA as per the manufacturer’s protocol. After addition of chromogenic substrate, the color change was examined by determining the absorbance at 450 nm using a microplate reader.

***Assessment of pro-inflammatory markers***

*HMGB1 and acHMGB1 levels*

Levels of sera HMGB1 and acHMGB1, as another important distinguishing feature of apoptotic versus necrotic cell death related to inﬂammation, were detected by ELISA kit according to the manufacturer’s instructions (23).

***Cytokines assessment in liver***

The assessment of cytokines in liver tissue was carried out in the protein extracts obtained by homogenization of liver tissue (0.5 mg of tissue/ml) in 50 mM Tris HCl, pH 7.4, 0.5 m M dithiothreitol and 10 µg/ml proteinase inhibitors, containing phenylmethylsulfonyl fluoride, pepstatin, and leupeptin. Levels of the cytokines, (IL-β and TNF-𝛼) were determined by a specific sandwich ELISA using capture/biotinylated detection.

***Measurement of hepatic myeloperoxidase (MPO) activity***

For the estimation of the MPO as a marker for neutrophil accumulation, MPO was extracted from the homogenates by freeze-thawing and sonication in 50 mM phosphate buffer (pH=6) containing 0.5% (w/v) hexadecyltrimethylammonium bromide, followed by centrifugation at 12,000 rpm for 15 min at 4 ^°^C. Then, 0.167 mg/ml *O*-dianisidine hydrochloride and 0.0005% (v/v) hydrogen peroxide in 50 mM phosphate buffer (pH=6) were added to an aliquot of the supernatant (24). The results were expressed as MPO U/mg protein, one unit of MPO activity was described as the amount that destroyed 1 μmol of H_2_O_2_

***Histopathological analysis***

Liver fragment tissues were fixed in 10 % formalin and processed by a standard method. Samples were sectioned (5-µm-thick), followed by staining with hematoxylin and eosin (H&E). 

***Statistical analysis***

Results were expressed as the mean±standard deviation (SD; n=12.). Data were analyzed using a one-way analysis of the variance test (One-way ANOVA) followed by the Honest significant difference test (HSD) of Tukey used as *post hoc* test to compare significance between groups at *P*<0.0*5* and *P*<0.01, using the Open stat 2014 program. 

## Results

***Phytochemical profiling***

*LC-MS/MS analysis*

**Figure 1:**
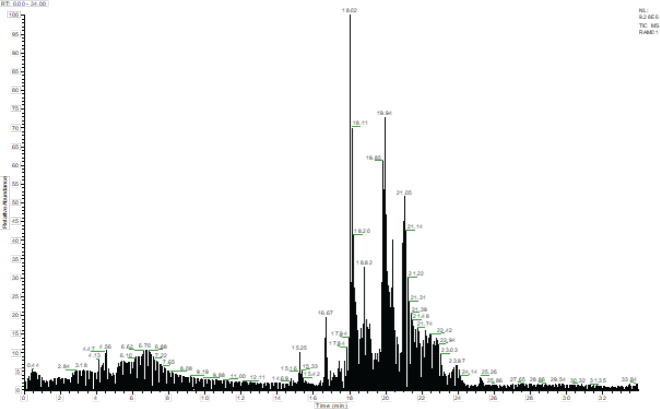
LC-MS/MS profile of *n-*BuOH extract of *Genista cephalantha* (BEGC)

The LC-MS/MS profile of BEGC is illustrated in Table 1 and Figure 1. Twelve phenolic compounds were recognized in the BEGC by comparing their retention times with those of obtainable commercial standards. The most abundant were *p*-hydroxy benzoïc acid (5810.130 mg/kg extract), *p*-coumaric acid (4371.809 mg/kg extract), rutin (2119.491), quercetin (1929.264 mg/kg extract), rosmarinic acid (1844.131 mg/kg extract), and caffeic acid (1791.046 mg/Kg), followed by 4OH-benzaldehit (1361.424 mg/Kg), naringenin (1147.077 mg/Kg), gentisic acid (379.536 mg/Kg), gallic acid (181.058), chlorogenic acid (84.584 mg/Kg), and oleuropein (29.433 mg/Kg).

***Acute toxicity of BEGC extract***

All rats in the treatment groups recorded normal behaviors and motors and no mortality was observed for administered BEGC extract. The rats were able to tolerate higher doses of BEGC. Therefore, the LD_50_ was assessed to be more than 2000 mg/kg for the extract. Thus, under these planned conditions, the dose of 200 mg/kg of BEGC was nominated for evaluation of hepatoprotective properties.

***Effect of BEGC on liver injury***

*Effect of BEGC on liver function*

**Figure 2:**
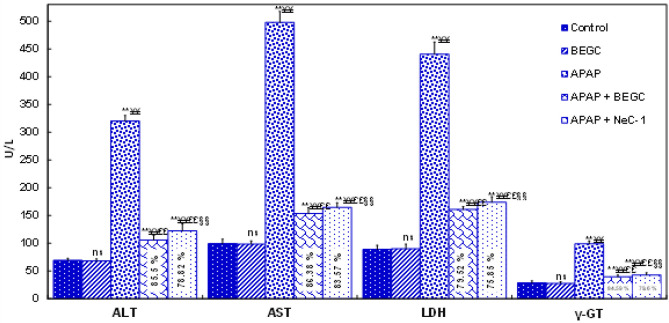
Effect of *Genista cephalantha* (BEGC)(200 mg/Kg) on hepatic markers (AST, ALT, LDH, and γ-GT activities)

Markers of liver function (AST, ALT, γ-GT, and LDH levels) increased significantly (*P*<0.01) in APAP-treated rats by approximately 5.006-fold, 4.61-fold, 3.4-fold, and 4.95-fold, respectively, compared to control (Figure 2). Pre-treatment with 200 mg/kg of BEGC significantly (*P*<0.01) restored hepatic marker enzymes (79.6-86.38%) in comparison with APAP-treated rats (Figure 2).

***Effect of BEGC on oxidative stress status***

*Effect of BEGC on ROS and MDA levels in intracellular and mitochondrial fractions*

**Figure 3:**
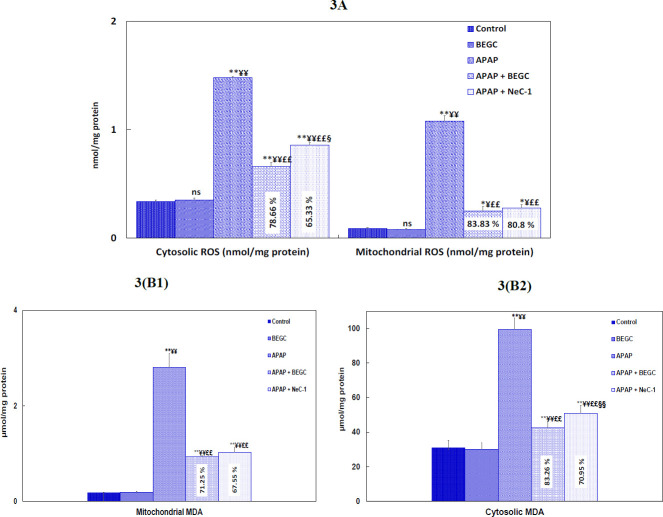
**A:** Effect of *Genista cephalantha* (BEGC)(200 mg/Kg) on cytosolic and mitochondrial ROS levels (nmol/mg protein)

Rats, treated with APAP, developed a significant increase (4.35-folds, 12-folds;* P*<0.01) in ROS generation in the cytosolic fraction and mitochondrial fraction of the liver (Figure 3 A). However, pretreatment with BEGC suppressed (78.66%, 83.83%,) cellular ROS levels in both cytosolic and mitochondrial fraction of liver in APAP-treated rats, respectively. Similarly, Nec-1 inhibited ROS production in the cytosolic and mitochondrial fractions (65. 33%, 80.8%) (Figure 3A). Moreover, pretreatment with BEGC can reduce up-regulation of MDA in the mitochondria (71.25 %; *P*<0.01)(Figure 3 (B1)) and in the cytosolic fractions (83.26%; *P*<0.01)(Figure 3 (B2)) of the liver in the APAP-group as compared to Nec-1 (67.55%, 70.95%; *P*<0.01) (Figure 3 (B1and B2)).

***Effect of BEGC on glutathione and glutathione-metabolizing enzymes in APAP-animals***

**Figure 4:**
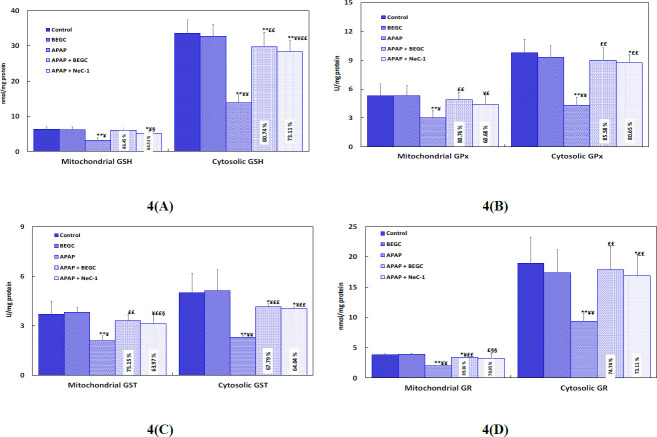
A, B, C, and D: Effect of *Genista cephalantha* (BEGC)(200 mg/Kg) on cytosolic glutathione and glutathione-metabolizing enzymes in liver tissues and on mitochondrial glutathione and glutathione-metabolizing enzymes against APAP-induced hepatotoxicity

In the present study, APAP treatment clearly depleted hepatic GSH and reduced GSH-related enzymes, as highlighted by decreased levels of GSH, GST, GR, and GPx in mitochondria and the cytosolic fraction of the liver. ((Figures 4 (A), 4(B), 4(C), and 4(D)). BEGC Treatment (200 mg/kg) effectively reserved the level of GSH and GSH-system enzymes towards normal levels in liver cytosolic and mitochondrial fractions (Figures (4(A), (4 (B) 4(C), and 4(D)). Pretreatment by BEGC restored GSH levels (80.74%; 86.45%; *P*<0.01) respectively in the cytosolic and mitochondrial liver fractions, demonstrating the comparable protective effect of BEGC and Nec-1 (73.11%, 64.511%; *P*<0.01) respectively (Figure 4 (A)). GPx activities (85.58%, 80.76%; *P*<0.01), GST activities (67.79%, 75.15%; *P*<0.01), and GR activities (89.16%, 74.07%; *P*<0.01) were significantly saved by prior administration of BEGC in hepatic cytosol and mitochondrial liver fractions (Figures 4 (B), 4 (C), and 4(D)). These altered levels in hepatic cytosol and mitochondrial fractions were also similarly regulated by pretreatment with Nec-1 (GPx 80.65%, 60.68%;* P*<0.01), GST (64.04%, 63.97%;* P*<0.01), and GR (78.95%, 66.66%; *P*<0.01), respectively (Figures 4 (B), (4 (C), and4(D)).

***Effect of BEGS on cell death markers***

*Effect of BEGS on caspase-3, caspase-8 activities, and CK 18 levels (M30, M65)*

**Figure 5:**
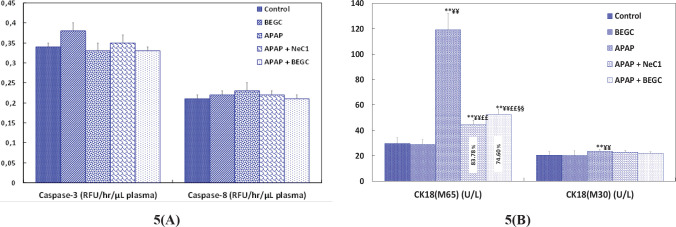
A: Effect of *Genista cephalantha* (BEGC)(200 mg/Kg) on plasma caspase-3 and caspase-8 levels (RFU/hr /µl) from rats exposed to APAP, B: Effect of BEGC (200 mg/Kg) on plasma CK 18 levels (M30, M65)(U/L) from rats exposed to APAP

Surprisingly, we observed that APAP treatment could not increase key indicators of apoptosis in either caspase 3 or caspase 8 (Figure 5A). Measurement of C K18 can indicate either a necrotic or apoptotic mode of cell death. More importantly, when we assessed the level of CK 18 levels (M30, M65), we found that concentration levels of the M65 antigen (full-length form of CK18; necrosis) were significantly (*P*<0.01)) greater in APAP rats and were approximately 4.04 times higher than the cleaved caspase form (ccCK18 fragments; M30; apoptosis)(Figure 5B), and no significant differences in M30 antigen concentrations were recorded between the two groups. This suggested that necrosis might play a more dominant role in cell death than apoptosis. However, pretreatment with BEGC extract significantly adjusted (74.60%; *P*<0.01) the alterations induced by APAP. A marked response of the M65 antigen level was observed in the Nec-1.-pretreatment (83.78%; *P*<0.01)(Figure 5B).

***Effect of BEGS on RIPK3 and MLKL levels***

**Figure 6:**
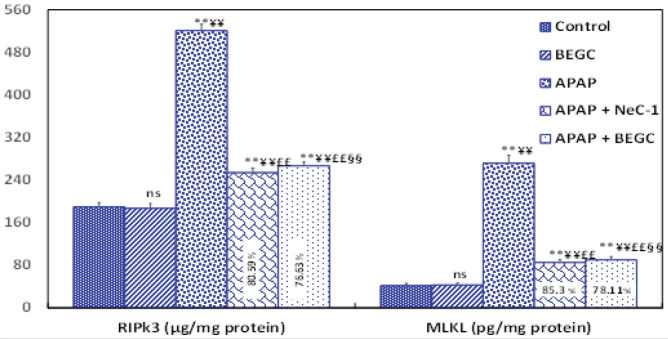
Effect of *Genista cephalantha* (BEGC)(200 mg/Kg) on necroptosis markers; RIPK3 (µg/mg protein) and MIKL (pg/mg protein) in liver tissues against APAP-induced hepatotoxicity

To explore the induction of hepatic necroptosis induced by APAP, we detected the levels of necroptosis-related proteins (RIPK3 and MLKL) in the livers at 72 hr after a single dose of APAP (1000 mg/kg). As shown in Figure 6, the level of RIPK3 and MLKL was significantly (*P*<0.01) enhanced (2.744-fold and 6.50-fold, respectively), in the group treated with APAP compared to the control group. These data suggest the possibility that RIPK3 may play a role in APAP-induced liver injury in rats and that RIPK-dependent necrosis is implicated in this model. These necroptotic factors (RIP3 and MLKL) were down-regulated significantly (76.63% and 78.11%;* P*<0.01, respectively) after BEGC pretreatment. A marked response was recorded with Nec-1 pretreatment (80.59% and 80.59%;* P*<0.01)(Figure 6).

***BEGC Reduces inflammation in APAP-Induced hepatotoxicity***

*Effect of BECS on pro-inflammatory cytokines in the liver*

**Figure 7:**
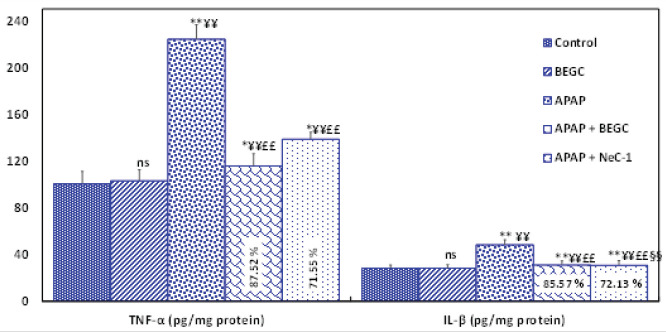
Effect of *Genista cephalantha* (BEGC)(200 mg/Kg) on proinflammatory markers TNF-α (pg/mg protein) and IL-β (pg/mg protein) in liver tissues against APAP-induced hepatotoxicity

As shown in Figure 7, administration of APAP, significantly (*P*<0.01) increases the pro-inflammatory cytokine production such as TNF-α (2.23-fold) and IL-β (1.77- fold) in the liver, however, BEGC pretreatment significantly (*P*<0.01*)* reduced the levels of TNF-𝛼 (87.52%) and IL-β (85. 57%) compared to Nec-1 (71.55% and 72.13%, respectively)(Figure 7).

***Effect of BEGC on HMGB1 levels***

**Figure 8:**
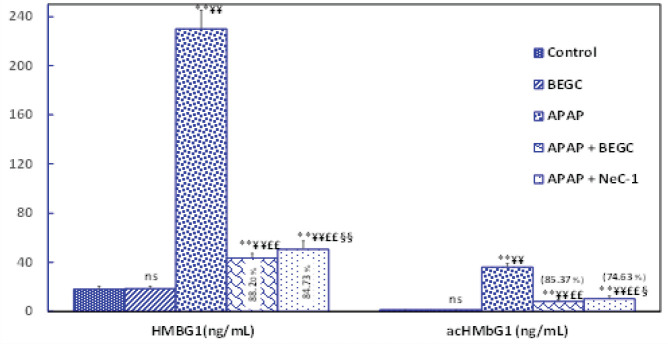
Effect of *Genista cephalantha* (BEGC)(200 mg/Kg) on sera levels of HMGB1 (ng/ml) and ac-HMGB1 (ng/ml) from rats exposed to APAP

Figure 8 illustrates that the sera levels of HMGB1 and ac-HMGB1 augmented significantly more than 12-fold and then 27-fold, compared to healthy controls within 72 hr of liver injury. However, BEGC administration significantly (*P*<0.01) reduced the levels of HMGB1 (88.20%) and ac-HMGB1 (85.37%) compared to Nec-1 (84.73% and 74.63 %, respectively)(Figure 8).

***Effect of BEGC on MPO activity in Liver tissues***

**Figure 9:**
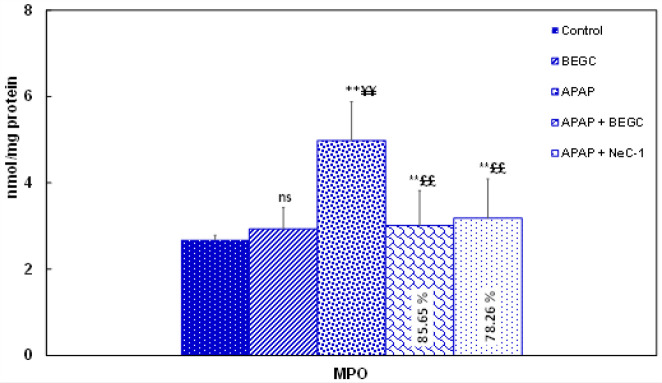
Effect of *Genista cephalantha* (BEGC)(200 mg/Kg) on MPO activity in liver tissues against APAP-induced hepatotoxicity

Neutrophil infiltration was assessed via MPO assessment. The increase in MPO activity reflects the massive recruitment of PMN in liver tissues (Figure 9). A significant (*P*<0.01) amplification was recorded in the MPO activity of the APAP group (4.98±0.9 U/mg protein) compared to control tissues (2.68±0.1 U/ mg of protein). However, BEGC pretreatment significantly ((85.65%); *P*<0.01) down-regulated MPO levels compared to Nec-1 (78.26%)(Figure 9).

***Histopathological examination***

**Figure 10:**
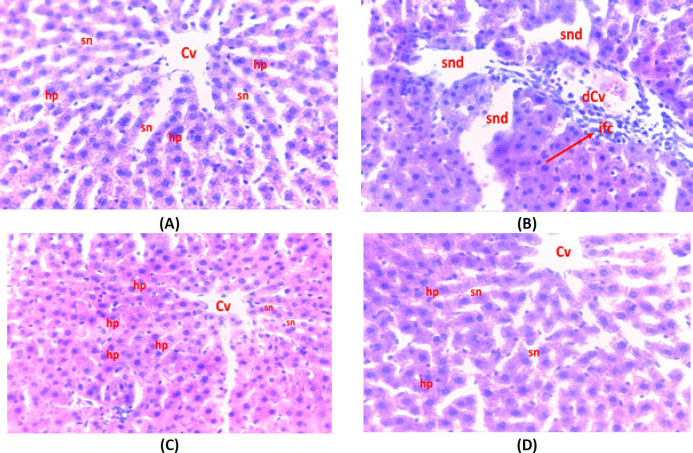
Micrograph of histopathological examination of liver tissues against APAP-induced hepatotoxicity (H&E, 400 xs)

The biochemical results were strongly supported by the results of the histopathological analysis. The histopathological report of the liver of APAP-treated rats showed severe changes in histoarchitecture, mixed inflammatory cell infiltration, parenchymal cell damage, and centrilobular necrosis as recorded in photomicrographs (Figure 10B). On the contrary, the liver injuries of rats pretreated with BEGC/or Nec-1 -1 were less severe and the hepatic architecture was reserved quite close to the liver of the control group (Figure 10 C, D).

## Discussion

The implication of ROS in APAP hepatotoxicity has been controversially discussed for decades, due to competing hypotheses, oxidant stress/LPO (25). Our results proved that APAP increases remarkably the mitochondrial and cytosolic levels of TBARS and ROS. On the mitochondrial level, the complexes of the respiratory chain I and III would be the primary sites of ROS production during programmed cell death (2). Free radical-mediated membrane lipid peroxidation may further have led to a lack of membrane integrity, leading to its rupture and subsequent release of cytosolic contents because of APAP-induced oxidative stress (26). Marked elevation of serum enzyme activities (AST, ALT, γ-GT, and LDH) indicated impaired liver functions and occurrence of hepatotoxicity (27). BEGC or NeC-1 pretreatment significantly abolished all these effects in APAP-exposed rats, decreasing the generation of TBARS and ROS through maintenance of liver function, indicating protection of the structural integrity of the hepatocyte cell membrane (28).

Extensive bioactivation of APAP has been reported to deplete the hepatic GSH pool and cause oxidative stress (29). Herein, APAP treatment induced a very significant reduction of overall hepatic GSH and mitochondrial GSH levels, impairment of the anti-oxidant defense system linked to GSH, and a subsequent weakening of the redox status. Since the liver performs a central role in GSH homeostasis between organs, liver disorders are expected to affect endogenous production and utilization of GSH, which in turn typically affects the glutathione system (30). The anti-oxidant effects of glutathione are immediately associated with GPx and GR, which may be key enzymes in the protection of redox homeostasis via protection against toxicity generated by free radicals (31). Cytosolic GSH depletion alone is not sufficient to determine lethal cell damage. There is evidence that activation of mitochondrial oxidative stress via NAPQI production in hepatocytes is a pivotal key in the mechanisms underlying APAP-mediated hepatotoxicity, mainly in mitochondria, resulting in mitochondrial dysfunction and cell death (27). Mitochondrial GSH (mGSH) is also considered the first line of defense in the mitochondrial membranes which allows the reduction of the hydro peroxidation and peroxidation on lipids and phospholipids via the actions of GSTs (32).

In our study, we were able to demonstrate the critical responses of the mitochondrial redox state to APAP-induced liver injury. In response, within the anti-oxidant system, depletion of mGSH level was followed by lower levels of mGPx and mGST enzymes. Taking into consideration that GPx1 is the fundamental isoform localized inside the cytosol, a smaller fraction can be present inside the mitochondrial matrix (33). 

Our findings revealed that the level of GSH and glutathione-metabolizing enzymes in liver homogenates and mitochondrial fractions were significantly saved by prior administration of BEGC or NeC-1. BEGC effects are more pronounced than those of NeC-1, which also up-regulated the glutathione system through ROS removal (34). BEGC efficiency may be explained by its potent anti-oxidant properties that enhance ROS elimination in hepatic cells. Indeed, several reviews have speculated the impact of flavonoids on the activation of the glutathione system (35). APAP cell death has been reported to be mediated by different modalities including apoptosis, autophagy, necrosis, and necroptosis (8). It was demonstrated that hepatocyte cell death pathways are mainly induced in APAP, in particular apoptosis or necrosis. This approach remains debatable and relative to the etiology duration and the expansion of liver damage (36). Diverse cell death pathways were indicated to be involved in hepatic damage caused by APAP, such as apoptosis (37), necrosis (38), and necroptosis (39). In the current study, to examine the type of APAP cell death, we established the markers of apoptosis (caspase activity and CK18) and necrosis (FK18 and HMGB1). To validate the apoptosis hypothesis of the cell death type, we first measured caspase activity. Most importantly, our findings revealed that no caspase-3 and -8 activities were detected after APAP administration in rat liver. To confirm these findings and to differentiate between the two different modes of cell death, we processed the protein keratin18 which is intensively synthesized in epithelial cells such as hepatocytes and plays a major role in the integrity of cell structure (40). Studies have shown that the full-length protein (fk18; M30) is substantially secreted by cells in a necrotic state, while caspase-cleaved keratin 18 (cck18; M65) is secreted after the induction of apoptosis (41). Given the fact that no apparent activation of caspase was detected in the APAP group and that fK18 levels in this group were higher; these findings suggest that necrosis is the main cause of APAP-induced cell death in the liver. 

Necroptosis, another form of cellular death, involves oligomerization of RIPK1 and RIPK3 leading to phospho-activation of MLKL by RIPK3 and has received much attention in recent years (42). Unlike apoptosis, necroptosis is a totally caspase-independent form of cell death (43). To verify the implication of RIPK-dependent necrosis in APAP-induced hepatocyte death in our model, we investigated whether BEGC could perturb hepatocyte death by regulating necroptosis. Our results revealed that the levels of RIPK3 and MLKL, mainly produced during necroptosis, are increased in the APAP group. Moreover, our results showed that necroptotic factors RIP3 and MLKL decreased in the liver after BEGC/Nec-1 pretreatment. Thus, BEGC which proved the same action as Nec-1, can be considered an anti-necrotic agent. Our results agree with many investigators who noted that APAP hepatotoxicity increases RIP3 expression (44, 13).

Another important characteristic of apoptotic cell death compared to necrotic cell death concerns inflammation (45). Apoptosis is known to cause little or no inflammation, while necrosis induces inflammation by releasing DAMPs, such as HMGB1 and IL-1 family cytokines (46). Furthermore, necroptosis is a non-apoptotic cell death mainly induced in inflammatory pathological conditions (47), it will therefore also be of interest to investigate whether BEGC engages pro-inflammatory inhibition in its protective action against APAP hepatotoxicity. Our findings demonstrated that total levels of HMGB1 and acHMGB1 were elevated in sera from APAP-overdosed rats with liver damage. In addition, our results revealed that APAP-liver damage was also associated with inflammation seen in the increasing levels of TNF-𝛼 and IL-β. In this regard, cytokines were proven to be crucial for the development of APAP-induced acute liver damage (48).

These previous constatations are consistent with the histological findings, revealing extensive neutrophil and Kupffer cell infiltration of the central vein peripherals and detectable loss of cellular boundaries in the APAP group reflecting rising MPO levels. Interestingly, both BEGC/Nec-1 successfully normalized hepatic markers of inflammation. Numerous necroptosis inhibitors have been studied for their impact on diverse human pathologies (49, 7).

Many necroptosis inhibitors from natural products have been tested; Xuan *et al* . showed that *Ganoderma lucidium* aqueous extract prevents necroptosis in brain cells of diabetic mice (50). Other researchers found that curcumin had neuroprotective effects by attenuating the necroptosis pathway (51). The biological properties shown by BEGC are in accord with several studies in the literature showing *Genista *species as candidate anti-oxidant and anti-inflammatory agents (52, 53). In our study, the hepatoprotective ability of BEGC may be explained by the abundance of polyphenols characterized by LC-MS/MS fingerprints; with a great presence of *p-*hydroxybenzoicacid, *p*-coumaric acid, rosmarinic acid, quercetin; naringenin, caffeic acid, and rutin. The potential synergistic effect of these phenolic compounds with respect to other less abundant phenolic compounds in *G. cephontala* (BEGC) possessing significant anti-oxidant properties, can be effective for the hepatoprotective activity of the studied plant (54, 55).

**Table 1:** LC-MS/MS profile of phenolic compounds from *n-*BuOH extract of *Genista cephalantha* (BEGC)

**N°**	**Compound name**	**MS/MS Ions studied**				**LOD** **(**mg/L)	**LOQ** **(**mg/L)	**RT** (min)	**Quantification** (mg phenolic/Kg Extract)
		**Parent** *(m/z*)	**fragments**(*m/z)*	**CE**	**Polarity**				
1	Gallic acid	169.7[M^-^H]^-^	80.5 126.2	25 16	- -	0.058	0.091	10.1	181.058
2	Gentisic acid	153.7[M^-^H]^-^	109.5	21	-	0.026	0.039	13.87	379.536
3	Chlorogenic acid	353.4[M^-^H]^-^	86.5 192.1	43 21	- -	0.051	0.072	14.25	84.584
4	*p*-Hydroxybenzoicacid	137.9[M^-^H]^-^	66.6 94.6	38 17	- -	0.243	0.519	14.64	5810.130
5	Caffeicacid	179.7[M^-^H]^-^	135.2 136.2	27 18	- -	0.042	0.058	15.26	1791,046
6	*p*-Coumaricacid	163.9[M^-^H]^-^	94.3 120.2	33 17	- -	0.069	0.109	16.97	4371.809
7	Rosmarinicacid	359.2[M^-^H]^-^	134.3 162.2	44 20	- -	0.029	0.050	17.86	1844.131
8	Naringenin	273[M^+^H]^+^	147.1 153	20 24	+ +	0.052	0.068	20.46	1147.077
9	Quercetin	301[M^-^H]^-^	152.1 179.9	23 20	- -	0.141	0.181	20.51	1929,264
10	4OH-benzaldehit	121[M^-^H]^-^	93.5 121.1	25 20	- -	0.032	0.059	15.32	1361.424
11	Rutin	609.4 [M^-^H]^-^	300.6 301.7	38 34	- -	0.022	0.034	18.04	2119,491
12	Oleuropein	121[M^-^H]^-^	93.5 121.5	25 20	- -	0.043	0.067	18.29	29.433

Parent: Precursor ion or Quasimolecular ion [M-H]- for ESI negative mode and [M+H]+ for ESI positive mode. CE: Collision energy in eV, RT: Retention time in minutes, LOD: Limit of detection, LOQ: Limit of quantification, (-): Negative polarity, (+): Positive polarity

Values are mean±SD (n=12), ns: not significant (comparison between controls) *: APAP group vs control groups; (**P<0.01). ¥: APAP group vs APAP+NeC-1 group; (^¥¥^*P<*0.01); £: APAP group vs APAP+BEGC group (^££^*P<*0.01); §: APAP+NeC-1 group vs APAP+BEGC group (^§§^*P<*0.01)

The % of protection is calculated as 100 x (values of APAP)-values of samples/(values of APAP)-values of control

AST: Aspartate-aminotransferase; ALT: Alanine aminotransferase; LDH: Lactate dehydrogenase; γ-GT: γ-glutamyl transferase; APAP: Acetaminophen

Values are mean±SD (n=12), ns: not significant (comparison between controls) *: APAP group vs control groups; (**P<0.01). ¥: APAP group vs APAP+NeC-1 group; (^¥¥^*P<*0.01); £: APAP group vs APAP+BEGC group (^££^*P<*0.01); §: APAP+NeC-1 group vs APAP+BEGC group (^§§^P<0.01). The % of protection is calculated as 100 x (values of APAP)-values of samples/(values of APAP)-values of control

**B1, B2:** Effect of BECM (200 mg/Kg) on the cytosolic and mitochondrial MDA levels (µmol/mg protein) in liver tissues against APAP-induced hepatotoxicity

Values are mean±SD (n=12), ns: not significant (comparison between controls) *: APAP group vs control groups; (**P<0.01). ¥: APAP group vs APAP+NeC-1 group; (^¥¥^*P<*0.01); £: APAP group vs APAP+BEGC group (^££^*P<*0.01); §: APAP+NeC-1 group vs APAP+BEGC group (^§§^*P<*0.01). The % of protection is calculated as 100 x (values of APAP)-values of samples/(values of APAP)-values of control

APAP: Acetaminophen; Nec-1: Necrostatin-1; MDA : Malondialdehyde

GSH (nmol/mg protein); GPx (U/mg protein); GST (U/mg protein), GR (U/mg protein). Values are mean±SD (n=12), ns: not significant (comparison between controls) *: APAP group vs control groups; (**P<0.01). ¥: APAP group vs APAP+NeC-1 group; (^¥¥^*P<*0.01); £: APAP group vs APAP+BEGC group (^££^*P<*0.01); §: APAP+NeC-1 group vs APAP+BEGC group (^§§^*P<*0.01)

The % of protection is calculated as 100 x (values of APAP)-values of samples/(values of APAP)-values of control

GSH: Reduced glutathione; GST: Glutathione;S-transferase; GPx: Glutathione peroxidase; GR: Glutathione reductase;APAP: Acetaminophen; Nec-1: Necrostatin-1

Values are mean±SD (n=12), ns: not significant (comparison between controls) *: APAP group vs control groups (**P<0.01). ¥: APAP group vs APAP+NeC-1 group (^¥¥^*P<*0.01); £: APAP group vs APAP+BEGC group (^££^*P<*0.01); §: APAP+NeC-1 group vs APAP+BEGC group (^§§^*P<*0.01)

The % of protection is calculated as 100 x (values of APAP)-values of samples/(values of APAP)-values of control

APAP: Acetaminophen; Nec-1: Necrostatin-1;fk18;M30: Cytokeratin 18 full length protein; cck18; M65 : caspase-cleaved keratin 18

Values are mean±SD (n=12), ns: not significant (comparison between controls); *: APAP group vs control groups (**P<0.01). ¥: APAP group vs APAP+NeC-1 group (^¥¥^*P<*0.01); £: APAP group vs APAP+BEGC group (^££^*P<*0.01); §: CP+NeC-1 group vs APAP+BEGC group (^§§^*P<*0.01)

The % of protection is calculated as 100 x (values of APAP)-values of samples/(values of APAP)-values of control

APAP: Acetaminophen; Nec-1: Necrostatin-1;RIPK3: Receptor-interacting protein kinase; MLKL: mixed lineage kinase domain-likeprotein

Values are mean±SD (n=12), ns: not significant (comparison between controls); *: APAP group vs control groups; (**P<0.01). ¥: APAP group vs APAP+NeC-1 group; (^¥¥^*P<*0.01); £: APAP group vs APAP+BEGC group (^££^*P<*0.01); §: APAP+NeC-1 group vs APAP+BEGC group (^§§^*P<*0.01)

The % of protection is calculated as 100 x (values of APAP)-values of samples/(values of APAP)-values of control

APAP: Acetaminophen; Nec-1: Necrostatin-1; TNF-α : Tumor necrosis factor-α; IL-β: Interleukin-β

Values are mean±SD (n=12), ns: not significant (comparison between controls) *: APAP group vs control groups (**P<0.01). ¥: APAP group vs APAP+NeC-1 group (^¥¥^*P<*0.01); £: APAP group vs APAP+BEGC group (^££^*P<*0.01); §: APAP+NeC-1 group vs APAP+BEGC group (^§§^*P<*0.01)

The % of protection is calculated as 100 x (values of APAP)-values of samples/(values of APAP)-values of control

APAP: Acetaminophen; Nec-1: Necrostatin-1; HMGB1: nuclear high mobility group box1; acHMGB1: Hyperacetylated

Values are mean±SD (n=12), ns: not significant (comparison between controls); *: APAP group vs control groups (**P<0.01). ¥: APAP group vs APAP+NeC-1 group (^¥¥^*P<*0.01)

The % of protection is calculated as: 100 x (values of APAP)-values of samples/(values of APAP)-values of control

APAP: Acetaminophen; Nec-1: Necrostatin-1; MPO : Myeloperoxidase

A. (Control-rats): normal cellular architecture with distinct hepatic cells(hp), showing a normal organization of hepatic cords near the central vein (Cv) with normal, sinusoidal spaces (sn) (Figure 10 (A))

B. (APAP-treated rats): The liver from the rat treated with APAP exhibited drastic alterations in histoarchitecture, liver construction was disrupted with damage to the radial arrangement of hepatic cords, hepatocellular disintegration was separated by dilated congested sinusoids (snd), a severe degree of hemorrhage (hr), irregularly dilated central vein (Cvd) with massive infiltration of inflammatory cells (ifc; arrow), centrilobular necrosis, and vacuolation of the pericentral vein hepatocytes (Figure 10(B))

C. (APAP + BEGC): BEGC showed notable recovery of APAP liver lesions compared to the control group. Minor deterioration of the liver parenchyma with a sufficient degree of histopathological alteration, moderate necrotic changes, reduced degree of sinusoidal and central vein enlargements, ballooning, and hepatocyte necrosis were noted (Figure 10(C))

D. (APAP + NeC-1): in the NeC-1 group, the hepatic architecture was preserved and showed regular hepatocyte cords, no pathological abnormalities were recorded, absence of vascular or necrosis changes, (Figure 10 (D))

APAP: Acetaminophen; Nec-1: Necrostatin-1

## Conclusion

Overall, the results presented here have proved, for the first time, that treatment with *n*-BuOH extract of *G.* *cephalenta* (BEGC) can prevent APAP-liver injury, which could be mainly attributed to multiple mechanisms, including maintenance of liver function, decrease in generation of TBARS and ROS, and restoration of GSH level and GSH system enzymes in mitochondrial and cytosolic fractions. BEGC was also successful in attenuating necrotic factors (fK18, RIPK3, and MLKL). Moreover, the effectiveness of BEGS pretreatment also appears to be mediated by regulating the production of pro-inflammatory agents (IL-β, TNF-α, MPO, HMGB1, acHMGB1). Interestingly, the hepatoprotective effect of BEGC which was comparable to Nec-1 in most parameters could be due to the abundance of polyphenols identified by LC-MS/MS analysis.

## Authors’ Contributions

B K performed all experiments. S A supervised and designed the study and assisted in all experiments. A M performed data analysis and statistics. R E performed the LC-MS/MS analysis. C K performed the extraction procedure. M R, S B, and F B supervised the phytochemical studies. All co-authors approve the current version of the manuscript. 

## Conflicts of Interest

The authors declare that they have no conflicts of interest.

## References

[1] Chiew AL, Gluud C, Brok J, Buckley NA (2018). Interventions for paracetamol (acetaminophen) overdose. Cochrane Database Syst Rev.

[2] Yan HM, Ramachandran A, Bajt ML, Lemasters JJ, Jaeschke H (2010). The oxygen tension modulates acetaminophen-induced mitochondrial oxidant stress and cell injury in cultured hepatocytes. Toxicol Sci.

[3] Malhi H, Gores GJ, Lemasters JJ (2006). Apoptosis and necrosis in the liver: A tale of two deaths. Hepatology.

[4] Jaeschke H, Bajt M L (2006). Intracellular signaling mechanisms of acetaminophen-induced liver cell death. Toxicol Sci.

[5] Galluzzi L, Kroemer G (2008). Necroptosis: A specialized pathway of programmed necrosis. Cell.

[6] Yan M, Huo Y, Yin S, Hu H (2018). Mechanisms of acetaminophen-induced liver injury and its implications for therapeutic interventions. Redox Biol.

[7] Cao L, Mu W (2020). Necrostatin-1 and necroptosis inhibition: Pathophysiology and therapeutic implications. Pharmacol Res.

[8] Vanden BT, Vanlangenakker N, Parthoens E, Deckers W, Devos M, Festjens N (2010). Necroptosis, necrosis and secondary necrosis converge on similar cellular disintegration features. Cell Death Differ.

[9] Quezel P, Santa S ( 1963). Nouvelle flore de l’Algérie et des régions désertiques méridionales.

[10] Chebbah K, Marchioni E, Menad A, Mekkiou R, Sarri D, Ameddah S (2014). Preliminary phytochemical screening, analysis of phenolic compounds and antioxidant activity of Genista cephalantha Spach (Fabaceae). Inter J phytomedicine.

[11] Kilkenny C, Browne WJ, Cuthill IC, Emerson M, Altman DG (2010). Improving bioscience research reporting: The arrive guidelines for reporting animal research. J Pharmacol Pharmacother.

[12] McGill MR, Williams CD, Xie Y, Ramachandran A, Jaeschke H (2012). Acetaminophen-induced liver injury in rats and mice: Comparison of protein adducts, mitochondrial dysfunction, and oxidative stress in the mechanism of toxicity. Toxicol Appl Pharmacol.

[13] Khodayar MJ, Kalantari H, Alidadi H, Khorsandi L, Ahangar N, Samimi A (2021). Taurine attenuates valproic acid-induced hepatotoxicity via modulation of RIPK1/RIPK3/MLKL-mediated necroptosis signaling in mice. Mol Biol Rep.

[14] Johnson D, Lardy H (1967). Isolation of liver or kidney mitochondria. Methods Enzymol.

[15] Gupta R, Dubey DK, Kannan GM, Flora SJ (2007). Concomitant administration of Moringa oleifera seed powder in the remediation of arsenic-induced oxidative stress in mouse. Cell Biol Inter.

[16] Ohkawa H, Ohishi N, Yagi K (1979). Assay for lipid peroxides in animal tissues by thiobarbituric acid reaction. Anal Biochem.

[17] Sedlak J, Hanus L (1982). Changes of glutathione and protein bound SH-groups concentration in rat adrenals under acute and repeated stress. Endocrinol Exp.

[18] Habig WH, Pabst MJ, Jakoby WB (1974). Glutathione S-transferases the first enzymatic step in mercapturic acid formation. J Biol Chem.

[19] Rotruck JT, Pope AL, Ganther HE, Swanson AB, Hafeman DG, Hoekstra WG (1973). Selenium: Biochemical role as a component of glutathione peroxidase. Science.

[20] Moss DW, Butterworth PJ ( 1974). Enzymology and Medicine.

[21] Lowry OH, Rosebrough NJ, Farr AL, Randall RJ (1951). Protein measurement with the Folin phenol reagent. J Biol Chem.

[22] Jiang W, Reich III CF, Pisetsky DS (2004). Mechanisms of activation of the RAW264 7 macrophage cell line by transfected mammalian DNA. Cell Immunol.

[23] Chen W, Bao G, Zhao L, Wang H (2020). Analysis of circulating HMGB1 in Human Serum. Methods Mol Biol.

[24] Bradley PP, Priebat DA, Christenses RD, Rothstein G (1982). Measurement of cutaneous inflammation: Estimation of neutrophil content with an enzyme marker. J Invest Dermatol.

[25] Ajiboye TO (2015). Standardized extract of Vitex doniana sweet stalls protein oxidation, lipid peroxidation and DNA fragmention in acetaminophen induced hepatotoxicity. J Ethnopharmacol.

[26] Ajiboye TO (2011). In vivo antioxidant potentials of Piliostigma thonningii (Schum) leaves: Studies on hepatic marker enzyme, antioxidant system, drug detoxifying enzyme and lipid peroxidation. Hum Exp Toxicol.

[27] Yuan L, Kaplowitz N (2013). Mechanisms of drug-induced liver injury. Clin Liver Dis.

[28] Davis M, Ideo G, Harrison NG, Williams R (1975). Hepatic glutathione depletion and impaired bromosulphthalein clearance early after paracetamol overdose in man and the rat. Clin Sci Mol Med.

[29] Ramachandran A, Lebofsky M, Baines CP, Lemasters JJ, Jaeschke H (2011). Cyclophilin D deficiency protects against acetaminophen-induced oxidant stress and liver injury. Free Radic Res.

[30] Abou Ghalia AH, Fouad IM (2000). Glutathione and its metabolizing enzymes in patients with different benign and malignant diseases. Clin Biochem.

[31] Hammond CL, Lee TK, Ballatori N (2001). Novel roles for glutathione in gene expression, cell death, and membrane transport of organic solutes. J Hepatol.

[32] Sims NR, Nilsson M, Muyderman H (2004). Mitochondrial glutathione: A modulator of brain cell death. J Bioenerg Biomembr.

[33] Arai M, Imai H, Koumura T, Yoshida M, Emoto K, Umeda M (1999). Mitochondrial phospholipid hydroperoxide glutathione peroxidase plays a major role in preventing oxidative injury to cells. J Biol Chem.

[34] Takemoto k, Hatano E, Iwaisako K, Takeiri M, Noma N, Ohmae S (2014). Necrostatin1protects against reactive oxygen species (ROS)-induced hepatotoxicity in acetaminophen-induced acute liver failure. FEBS Open Bio.

[35] Durgo K, Vukovic L, Rusak G, Osmak M, Colic JF (2007). Effect of flavonoids in glutathione level, lipid peroxidation and cytochrome P450 CYP1A1 expression in human Laryngeal carcinoma cells. Food Technol Biotechnol.

[36] Jaeschke H, McGill MR, Ramachandran A (2012). Oxidant stress, mitochondria, and cell death mechanisms in drug-induced liver injury: Lessons learned from acetaminophen hepatotoxicity. Drug Metab Rev.

[37] Jaeschke H, Duan L, Akakpo JY, Farhood A, Ramachandran A (2018). The role of apoptosis in acetaminophen hepatotoxicity. Food Chem Toxicol.

[38] Gujral JS, Knight TR, Farhood A, Bajt ML, Jaeschke H (2002). Mode of cell death after acetaminophen overdose in mice: Apoptosis or oncotic necrosis?. Toxicol Sci.

[39] Zhang YF, He W, Zhang C, Liu XJ, Lu Y, Wang H (2014). Role of receptor interacting protein (RIP)1 on apoptosis-inducing factor-mediated necroptosis during acetaminophen-evoked acute liver failure in mice. Toxicol Lett.

[40] Kramer G, Erdal H, Martens HJ, Nap M, Mauermann J, Steiner G (2004). Differentiation between cell death modes using measurements of different soluble forms of extracellular cytokeratin 18. Cancer Res.

[41] Antoine DJ, Williams DP, Kipar A, Jenkins RE, Regan SL, Sathish JG (2009). High-mobility group box-1 protein and keratin-18, circulating serum proteins informative of acetaminophen-induced necrosis and apoptosis in vivo. Toxicol Sci.

[42] Jia Y, Feixia W, Qin G, Mengmeng L, Ling W, Zili Z (2018). Curcumol induces RIPK1/RIPK3 complex-dependent necroptosis via JNK1/2-ROS signaling in hepatic stellate cells. Redox Biol.

[43] Deutsch M, Graffeo CS, Rokosh R, Pansari M, Ochi A, Levie EM (2015). Divergent eﬀects of RIP1 or RIP3 blockade in murine models of acute liver injury. Cell Death Dis..

[44] Ramachandran A, McGill MR, Xie Y, Ni HM, Ding WX, Jaeschke H (2013). Receptor interacting protein kinase 3 is a critical early mediator of acetaminophen-induced hepatocyte necrosis in mice. Hepatology.

[45] Pasparakis M, Vandenabeele P (2015). Necroptosis and its role in inflammation. Nature.

[46] Lawson JA, Farhood A, Hopper RD, Bajt ML, Jaeschke H (2000). The hepatic inflammatory response after acetaminophen overdose: Role of neutrophils. Toxicol Sci.

[47] Lau A, Wang S, Jiang J, Haig A, Pavlosky A, Linkermann A (2013). RIPK3-mediated necroptosis promotes donor kidney inflammatory injury and reduces allograft survival. Am J Transplant.

[48] Jaeschke H (2018). Mechanisms of sterile inflammation in acetaminophen hepatotoxicity. Cell Mol Immunol.

[49] Degterev A, Ofengeim D, Yuan J (2019). Targeting RIPK1 for the treatment of human diseases. Proc Natl Acad Sci U S A.

[50] Xuan M, Okazaki M, Iwata N, Asano S, Kamiuchi S, Matsuzaki H (2015). Chronic treatment with a water-soluble extract from the culture medium of ganoderma lucidum mycelia prevents apoptosis and necroptosis in hypoxia/ischemia-induced injury of type 2 diabetic mouse brain. Evid Based Complement Alternat Med.

[51] Dai MC, Zhonga ZH, Suna YH, Suna QF, Wang YT, Yanga GY (2013). Curcumin protects against iron induced neurotoxicity in primary cortical neurons by attenuating necroptosis. Neurotox Res.

[52] Baali N, Belloum Z, Baali S, Chabi B, Pessemesse L, Fouret G (2016). Protective activity of total polyphenols from Genista quadriflora Munby and Teucrium polium geyrii Maire in acetaminophen-induced hepatotoxicity in rats. Nutrients.

[53] Ait-kaciAourahoun K, Fazouane F, Benayache S, Bettache Z, Benayad T, Denni N (2019). Antioxidant and anti-inflammatory activity of phenolic extracts of Genista ferox (Fabaceae) Pak. J Pharm Sci.

[54] Abarikwu SO, Njoku RC, John IG, Amadi BA, Mgbudom-Okah CJ, Onuah CL (2022). Antioxidant and anti-inflammatory protective effects of rutin and kolaviron against busulfan-induced testicular injuries in rats. Syst Biol Report Med.

[55] Skroza D, Simat V, Vrdolj L (2022). Investigation of antioxidant synergisms and antagonisms among phenolic acids in the model matrices using FRAP and ORAC methods. Antioxidants.

